# Hemilaminectomy versus laminoplasty for intradural extramedullary tumors: A comparison of postoperative axial pain and regional outcomes

**DOI:** 10.1016/j.bas.2026.106044

**Published:** 2026-04-10

**Authors:** Joo-Sung Kim, Jiwon Woo, Dongkyu Kim, Bong-Ju Moon, Kyung-Hyun Kim, Jeong-Yoon Park, Sung-Uk Kuh, Dong-Kyu Chin, Keun-Su Kim, Hyun-Jun Jang

**Affiliations:** a Department of Neurosurgery, Spine and Spinal Cord Institute, Severance Hospital, Yonsei University College of Medicine, Seoul, South Korea; b Department of Neurosurgery, Spine and Spinal Cord Institute, Gangnam Severance Hospital, Yonsei University College of Medicine, Seoul, South Korea

**Keywords:** Spinal cord tumor, Intradural extramedullary tumor, Minimal invasive spine surgery, Axial pain, Hemilaminectomy

## Abstract

**Objective:**

Intradural extramedullary (IDEM) tumor surgery traditionally focuses on gross total resection (GTR), but postoperative quality of life—specifically chronic axial pain—has emerged as a critical metric. Conventional laminoplasty, despite being restorative, involves bilateral muscle stripping that may cause persistent axial morbidity.

**Research question:**

This study investigated whether the unilateral, muscle-preserving approach of hemilaminectomy translates into superior long-term axial pain control compared to laminoplasty.

**Methods:**

We retrospectively reviewed 99 patients (Hemilaminectomy: 55; Laminoplasty: 44). To isolate the impact of surgical trauma, cases with significant facet violations or pre-existing instability were excluded. Axial and radiating pain (NRS) were assessed at 1, 6, and 12 months postoperatively.

**Results:**

Preoperative disc and facet degeneration were comparable between groups (p > 0.05). While radiating pain relief was equivalent, the hemilaminectomy group showed significantly lower axial pain at 6 months (1.54 ± 1.89 vs. 2.58 ± 1.79, p = 0.002) and 1 year (2.07 ± 2.06 vs. 2.63 ± 1.70, p = 0.049). This benefit was most pronounced in the thoracic subgroup, which showed superior 6-month axial pain control (p = 0.002) and continuous recovery from 1 month to 1 year (p = 0.028) compared to the laminoplasty group.

**Conclusion:**

Hemilaminectomy provides a distinct minimally invasive advantage by preserving the posterior midline structures and contralateral muscles. This structural integrity leads to a significant reduction in chronic surgery-induced axial pain compared to laminoplasty, particularly in the thoracic spine.

## Highlights

• Hemilaminectomy reduces chronic axial pain compared to laminoplasty for IDEM tumors.
• The benefit of hemilaminectomy is most significant in the thoracic region.
• Both techniques achieve equivalent radiating pain relief and tumor resection.
• Baseline degeneration was matched to isolate the impact of surgical approaches.
• Preserving midline structures is crucial for preventing surgery-induced axial pain.

## 1 Introduction

Surgical management of intradural extramedullary (IDEM) tumors has traditionally focused on achieving gross total resection (GTR) while minimizing neurological morbidity (Seppälä et al., 1995; Piñeiro et al., 2025; Liao et al., 2023). With advancements in microsurgical techniques and intraoperative monitoring, GTR has become a predictable outcome (Piñeiro et al., 2025; Liao et al., 2023). Recently, the paradigm of spinal tumor surgery has been continuously evolving towards minimizing approach-related morbidity, and for instance, the application of robotic-assisted systems, has been successfully introduced for minimally invasive tumor resections in the thoracic and lumbosacral regions (Choucha et al., 2025). However, as survival rates and functional successes improve, postoperative quality of life—specifically chronic axial pain—has emerged as a critical patient-centered metric that often determines the overall success of the procedure.

While total laminectomy has been the traditional approach, the associated extensive muscle denervation and disruption of the posterior ligamentous complex (PLC) often result in chronic postoperative axial pain (He et al., 2020). These structural alterations often lead to “post-laminectomy syndrome,” characterized by persistent axial pain (Christelis et al., 2021). Laminoplasty was developed as an alternative, yet it still involves bilateral muscle stripping and potential damage to the spinous process-ligamentous complex.

The mechanism of pain in these patients evolves through the perioperative period. Preoperative radiating pain results from tumor-induced nerve root compression, and preoperative axial pain stems from the tumor's mass effect. However, persistent postoperative axial pain is largely attributed to surgical trauma—specifically the iatrogenic injury to muscles and ligaments—rather than the tumor itself (Schönnagel et al., 2024; Wang et al., 2011). While randomized controlled trials (RCTs) have shown that hemilaminectomy is equivalent to total laminectomy in terms of resection rates and operative efficiency, its value as a minimally invasive technique deserves further attention (Piñeiro et al., 2025). In segments without pre-existing degenerative lesions, preserving the midline structures and contralateral soft tissues may be the key to preventing long-term axial morbidity. The thoracic spine may be more sensitive to surgical trauma due to its relatively preserved state compared to the highly mobile cervical and lumbar regions.

This study investigated whether this structural preservation translates into a reduction in postoperative axial pain across different spinal regions. By comparing hemilaminectomy and laminoplasty, we sought to identify the specific spinal regions that derive the greatest benefit from this minimally invasive technique and facilitate continuous functional recovery.

## 2 Methods

### 2.1 Study population

This retrospective cohort study received approval from the Institutional Review Board of our hospital, and the requirement for informed consent was waived. We initially identified patients who underwent surgical resection for IDEM tumors at our center between January 2023 and December 2024. To precisely evaluate surgery-induced axial pain in segments without pre-existing degenerative lesions, we strictly excluded patients with dumbbell-type tumors (Eden type II, III, or IV), iatrogenic facet joint violation exceeding 50%, or those requiring total facetectomy for tumor exposure. Patients with pre-existing spinal instability or those undergoing concurrent fusion procedures were also excluded to ensure the analysis focused solely on the impact of the primary access techniques. The choice of surgical approach — hemilaminectomy or laminoplasty — was primarily determined by the operating surgeon's preference. All procedures were performed by four fellowship-trained spine surgeons, each with more than 10 years of experience. As a general tendency, two surgeons preferentially performed laminoplasty, while the other two favored hemilaminectomy. Laminoplasty was preferentially selected for tumors located at or near the spinal midline, including en plaque meningiomas, for which determining the optimal laterality for a hemilaminectomy approach is technically challenging and wider exposure is considered necessary for safe resection.

### 2.2 Surgical technique: unilateral hemilaminectomy (Fig. 1)

The hemilaminectomy was performed using a focused unilateral approach. A limited midline skin incision was followed by subperiosteal muscle dissection restricted to the symptomatic side. This technique allowed for a surgical corridor while meticulously preserving the midline PLC and the contralateral paraspinal musculature. Under microscopic guidance, a high-speed drill was used to perform the hemilaminectomy, ensuring the base of the spinous process remained intact to maintain structural continuity with the contralateral side. For tumors with ventral or ventrolateral extensions, the operating table was rotated to optimize visualization and facilitate safe excision without spinal cord manipulation. After GTR, the dura was closed in a watertight fashion and reinforced with fibrin sealant.

### 2.3 Surgical technique: laminoplasty with reconstruction (Fig. 2)

Laminoplasty involved a bilateral posterior midline approach to provide a comprehensive surgical field. Following bilateral subperiosteal elevation of the paraspinal muscles, gutters were created at the lamina-facet junction using a high-speed drill to facilitate an en bloc laminotomy. The entire posterior laminar arch was removed as a single unit for microscopic tumor resection. Once GTR and dural closure were finalized, the removed posterior arch was repositioned to its original anatomical site to restore the spinal canal roof. The arch was rigidly secured using a titanium mini-plate system fixed with micro-screws (Lorenz Plating System; Walter Lorenz Surgical, Jacksonville, FL, USA). This anatomical restoration provided a protective framework for the neural elements and a stable substrate for the reattachment of the bilaterally striped muscles.

### 2.4 Radiological assessment of spinal degeneration

To evaluate preoperative baseline spinal conditions, disc and facet joint degeneration were assessed at the surgical levels using preoperative Magnetic Resonance Imaging (MRI) and Computed Tomography (CT) scans. Disc degeneration was evaluated on T2-weighted sagittal MRI and graded from 0 to 5 according to the Pfirrmann classification system (Pfirrmann et al., 2001). Facet joint degeneration was assessed on axial CT scans and graded from 0 to 3 based on the Weishaupt grading system, which considers joint space narrowing, sclerosis, and osteophyte formation (Weishaupt et al., 1999). All radiological evaluations were performed by two independent observers blinded to the surgical approach, and the mean scores were used for analysis to ensure group homogeneity and to investigate the impact of pre-existing degeneration on postoperative axial pain.

### 2.5 Clinical assessment and statistical analysis

Postoperative outcomes were evaluated using the Numeric Rating Scale (NRS) for axial (neck/back) and radiating (arm/leg) pain. Scores were recorded preoperatively and at 1-month (1M), 6-month (6M), and 1-year (1Y) follow-up intervals. All patients were recommended to wear a postoperative brace until 1M, and appropriate analgesics, such as non-steroidal anti-inflammatory drugs or acetaminophen, were concurrently prescribed. Statistical analysis was performed using Python (version 3.13) with the Pandas, SciPy, and NumPy libraries. To compare continuous variables such as NRS scores and patient age, the Mann-Whitney *U* test was employed, while categorical data were analyzed via the Chi-square or Fisher's exact test. Subgroup analyses were further conducted for the Cervical, Thoracic, and Lumbar regions to assess level-specific recovery patterns. All data are presented as mean ± standard deviation (SD), and statistical significance was set at p < 0.05.

## 3 Results

### 3.1 Baseline characteristics and surgical outcomes (Table 1)

The study included 99 patients: 55 in the hemilaminectomy group and 44 in the laminoplasty group. There were no statistically significant differences between the two groups regarding age (52.1 ± 15.6 vs. 53.0 ± 17.5 years; p = 0.923), sex distribution (p = 0.842), or pathological diagnosis (p = 0.115). The mean number of surgical segments was 1.98 ± 0.49 in the hemilaminectomy group and 2.18 ± 1.32 in the laminoplasty group (p = 0.987). Total resection was achieved in 100% of the hemilaminectomy group. In the laminoplasty group, total resection was achieved in all cases except for three cases of en plaque or broad-based meningiomas.

### 3.2 Radiological evaluation: disc and facet degeneration (Table 2)

Preoperative radiological parameters showed no significant differences between the surgical groups for overall disc degeneration (1.44 ± 1.29 vs. 1.77 ± 1.48; p = 0.300) or facet degeneration (0.69 ± 0.84 vs. 0.82 ± 0.97; p = 0.618). Comparison across spinal regions using the Kruskal-Wallis test showed a significant difference in facet degeneration (p = 0.044). Post-hoc analysis (Dunn's test) revealed that the thoracic level had lower facet degeneration scores (0.59 ± 0.80) compared to the lumbar level (1.09 ± 1.03; p = 0.018). Disc degeneration scores did not show significant regional differences (p = 0.337).

### 3.3 Longitudinal clinical outcomes: overall axial and radiating pain (Table 3, Fig. 3)

Axial and radiating pain scores decreased in both groups from preoperative levels through the 1-year follow-up. For radiating pain, no significant differences were observed between the two groups at any follow-up interval (p > 0.05). Regarding axial pain, the hemilaminectomy group had lower NRS scores at 6 months (1.54 ± 1.89 vs. 2.58 ± 1.79; p = 0.002) and 1 year (2.07 ± 2.06 vs. 2.63 ± 1.70; p = 0.049) compared to the laminoplasty group. The change in axial pain from 1 month to 1 year (Delta 1M-1Y) was 0.93 ± 1.77 in the hemilaminectomy group and −0.17 ± 1.65 in the laminoplasty group (p = 0.007).

### 3.4 Subgroup analysis: clinical outcomes by spinal region (Fig. 4)

Subgroup analysis was performed for the cervical (n = 17), thoracic (n = 51), and lumbar (n = 31) regions. Within each subgroup, no significant differences were found between the hemilaminectomy and laminoplasty groups in terms of age, sex distribution, or the number of surgical segments (p > 0.05 for all parameters, Table 4).

For the cervical and lumbar subgroups, both the hemilaminectomy and laminoplasty groups showed significant postoperative improvement in axial and radiating pain compared to preoperative levels. However, no statistically significant differences were observed between the two surgical groups at any follow-up time point (1M, 6M, and 1Y) for either axial or radiating pain in these regions (all p > 0.05).

In the thoracic subgroup, the hemilaminectomy group showed lower axial NRS scores at 6 months (1.15 ± 1.46 vs. 2.90 ± 1.89; p = 0.002) and greater pain reduction from 1 month to 1 year (0.75 ± 1.86 vs. −0.62 ± 1.66; p = 0.028). Regarding radiating pain, however, no significant differences were observed between the hemilaminectomy and laminoplasty groups regarding radiating pain at any follow-up interval (Table 5, Fig. 4).

**Fig. 1:**
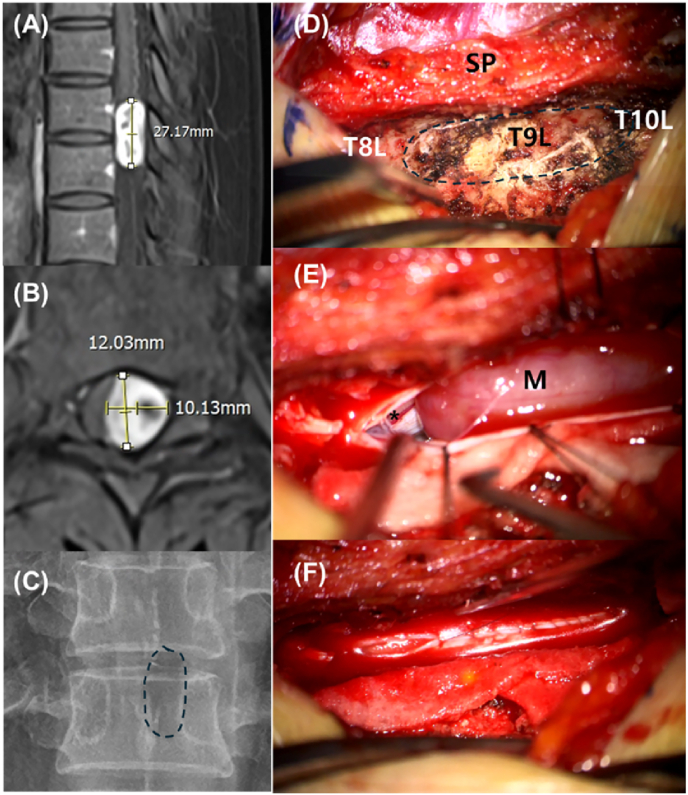
Illustrative case of a T9–10 intradural extramedullary tumor resection in a 39-year-old female. (A) Preoperative sagittal T1-weighted gadolinium-enhanced MRI showing a well-defined enhancement of the tumor mass at the T9–10 level, measuring 27 ∗ 12 ∗ 10 mm (height ∗ depth ∗ width). (B) Preoperative axial T1-weighted gadolinium-enhanced MRI demonstrating the intradural mass causing significant compression of the spinal cord. (C) Postoperative radiograph (X-ray). The dotted circle indicates the extent of the hemilaminectomy. (D) Intraoperative microscopic view following unilateral exposure. The right-side fascia remains intact and is retracted to the right. (SP: spinous process; T8L, T9L, and T10L: left laminae of T8, T9, and T10, respectively). (E) Intraoperative microscopic view after dural opening. The tumor mass (M) is clearly exposed, showing the severely compressed spinal cord (indicated by an asterisk, ∗). (F) Intraoperative microscopic view during dural closure. A watertight primary suture of the dura mater is performed to prevent cerebrospinal fluid leakage.

**Fig. 2:**
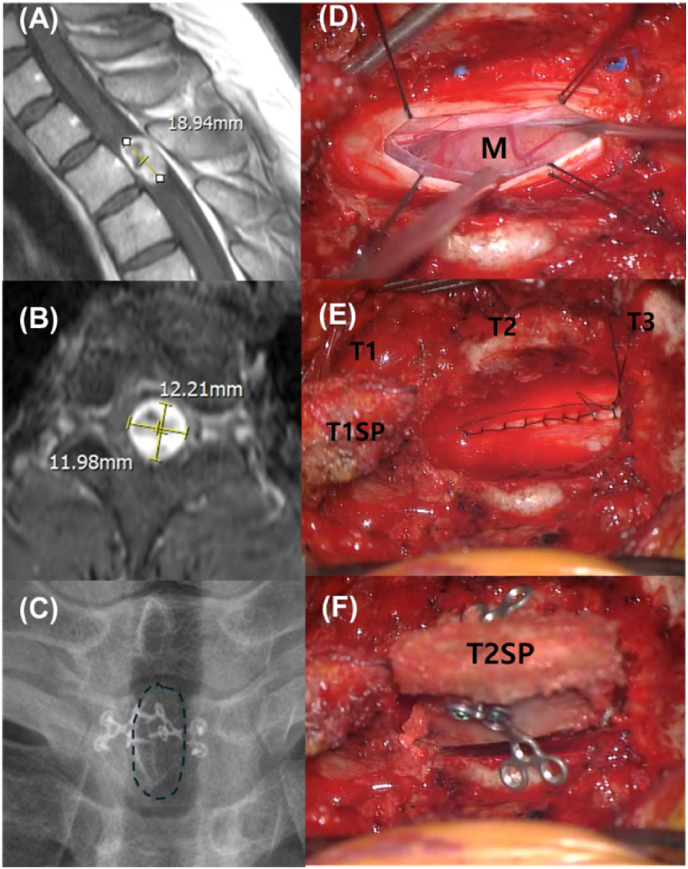
Surgical resection of an intradural extramedullary tumor at the T2 level in a 42-year-old male using the laminoplasty technique. (A) Preoperative sagittal T1-weighted gadolinium-enhanced MRI showing an IDEM tumor at the T2 level, measuring 18 ∗ 12 ∗ 12 mm (height ∗ depth ∗ width). (B) Preoperative axial T1-weighted gadolinium-enhanced MRI demonstrating significant spinal cord compression by the intradural mass. (C) Postoperative radiograph (X-ray). The dotted circle indicates the extent of the laminoplasty. (D) Intraoperative microscopic view after dural opening. The tumor mass (M) is clearly exposed and separated from the neural tissues. (E) Intraoperative microscopic view after watertight dural suture. The laminae of T1, T2, and T3, and the T1 spinous process (T1SP) are identified. (F) Intraoperative microscopic view during reconstruction. The T2 spinous process (T2SP) is being repositioned and secured using a titanium plate and screws to restore the posterior column.

**Fig. 3:**
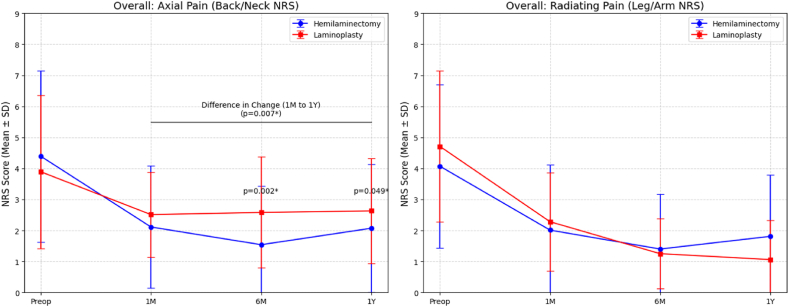
Longitudinal changes in Numeric Rating Scale (NRS) scores for axial and radiating pain.

**Fig. 4:**
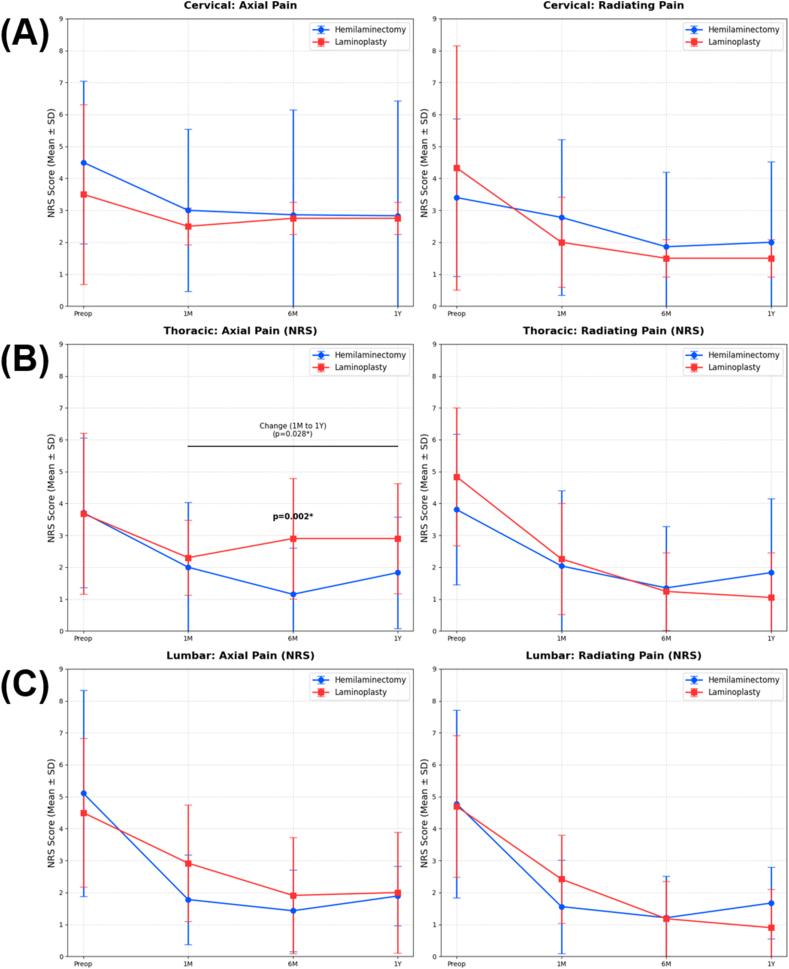
Subgroup analysis of Numeric Rating Scale (NRS) for axial and radiating pain categorized by spinal regions (Cervical, Thoracic, and Lumbar).

## 4 Discussion

The primary objective of this study was to compare the clinical outcomes of unilateral hemilaminectomy and laminoplasty for the resection of IDEM tumors, with a specific focus on postoperative axial pain across different spinal regions (Piñeiro et al., 2025). Our results demonstrate that while both techniques are effective for tumor removal, hemilaminectomy offers a significant advantage in reducing long-term axial pain, particularly in the thoracic spine. Since GTR was achieved in nearly all patients, the influence of the tumor on axial pain was effectively eliminated postoperatively. Consequently, postoperative pain is primarily driven by two factors: pre-existing spinal degeneration and the surgical approach. Our analysis showed no significant differences in preoperative disc or facet degeneration between the two groups (Table 2). By matching these baseline variables, we could isolate the surgical approach as the primary factor affecting long-term axial morbidity.

**Table 1:** Patient baseline characteristics and surgical outcomes.

	Hemilaminectomy (N = 55)	Laminoplasty (N = 44)	p-value
Age (years)	52.1 ± 15.9	53.0 ± 18.3	0.800
Sex (Female)	33 (60.0%)	27 (61.4%)	1.000
Tumor Level			0.632
Cervical	10 (18.2%)	7 (15.9%)	
Thoracic	26 (47.3%)	25 (56.8%)	
Lumbar	19 (34.5%)	12 (27.3%)	
Pathology			0.139
Schwannoma	35 (63.6%)	19 (43.2%)	
Meningioma	17 (30.1%)	21 (47.7%)	
Others	4 (7.3%)	4 (9.1%)	
Surgery segments	1.98 ± 0.49	2.18 ± 1.32	0.712

Values are presented as mean ± standard deviation (SD), or number (%).

**Table 2:** Preoperative radiological characteristics: Disc and facet degeneration.

		Hemilaminectomy (N = 55)	Laminoplasty (N = 44)	p-value
Disc Degeneration	Overall	1.44 ± 1.29	1.77 ± 1.48	0.300
	Cervical (A)	1.70 ± 1.34	2.33 ± 1.63	0.439
	Thoracic (B)	1.12 ± 0.99	1.60 ± 1.29	0.222
	Lumbar (C)	1.74 ± 1.56	1.85 ± 1.77	1.000
Facet Degeneration	Overall	0.69 ± 0.84	0.82 ± 0.97	0.618
	Cervical (A)†	0.40 ± 0.70	0.83 ± 0.75	0.223
	Thoracic (B) †∗	0.50 ± 0.71	0.68 ± 0.90	0.517
	Lumbar (C) †∗	1.11 ± 0.94	1.08 ± 1.19	0.794

Values are presented as Mean ± Standard deviation (SD). Disc degeneration was assessed using the Pfirrmann classification (0–5); Facet joint degeneration was assessed using the Weishaupt grading system (0–3). †Kruskal-Wallis test revealed a significant difference in facet degeneration across the three spinal levels (p = 0.044). ∗Post-hoc analysis (Dunn's test) showed that Thoracic (B) segments had significantly lower facet degeneration than Lumbar (C) segments (p = 0.018).

**Table 3:** Longitudinal clinical outcomes for overall axial and radiating pain.

NRS	Hemilaminectomy (N = 55)	Laminoplasty (N = 44)	p-value
Axial pain			
Preop	4.39 ± 2.76	3.89 ± 2.47	0.603
POD 1M	2.11 ± 1.97	2.51 ± 1.37	0.169
POD 6M	1.54 ± 1.89	2.58 ± 1.79	0.002∗
POD 1Y	2.07 ± 2.06	2.63 ± 1.70	0.049∗
Delta (Pre-1M)	2.35 ± 3.22	1.63 ± 2.34	0.181
Delta (Pre-6M)	2.67 ± 3.41	1.36 ± 2.75	0.086
Delta (Pre-1Y)	2.38 ± 3.71	1.46 ± 2.71	0.332
Delta (1M-1Y)	0.93 ± 1.77	−0.17 ± 1.65	0.007∗
Radiating pain			
Preop	4.08 ± 2.61	4.71 ± 2.44	0.167
POD 1M	2.00 ± 2.11	2.28 ± 1.57	0.213
POD 6M	1.39 ± 1.79	1.25 ± 1.13	0.681
POD 1Y	1.81 ± 1.98	1.06 ± 1.26	0.087
Delta (Pre-1M)	2.23 ± 3.30	2.20 ± 2.55	0.835
Delta (Pre-6M)	2.67 ± 3.44	3.18 ± 2.33	0.582
Delta (Pre-1Y)	2.35 ± 3.68	3.54 ± 2.63	0.185
Delta (1M-1Y)	1.19 ± 1.47	1.29 ± 1.71	0.651

Values are presented as Mean ± standard deviation (SD). NRS, Numeric Rating Scale; Preop, preoperative; 1-month, 6-months, 1-year postoperatively (1M/6M/1Y).Delta 1M–1Y represents the change in NRS scores from 1 month to 1 year postoperatively.∗Statistically significant difference between groups (p < 0.05).

**Table 4:** Patient characteristics across spinal region subgroups.

		Hemilaminectomy (N = 55)	Laminoplasty (N = 44)	p-value
Cervical (n = 17)	Age (years)	46.4 ± 21.5	56.4 ± 16.2	0.525
	Sex (Male: Female)	4 : 6	3 : 4	1
	Surgical Segments	2.10 ± 0.32	3.14 ± 2.67	0.395
Thoracic (n = 51)	Age (years)	53.2 ± 14.9	54.7 ± 15.2	0.792
	Sex (Male: Female)	12 : 14	8 : 17	0.454
	Surgical Segments	2.00 ± 0.63	2.04 ± 0.84	0.967
Lumbar (n = 31)	Age (years)	53.7 ± 14.0	47.4 ± 24.7	0.685
	Sex (Male: Female)	6 : 13	6 : 6	0.518
	Surgical Segments	1.89 ± 0.32	1.75 ± 0.75	0.358

Values are presented as Mean ± SD or Number (%).

**Table 5:** Longitudinal clinical outcomes across spinal region subgroups.

	NRS	Hemilaminectomy (N = 55)	Laminoplasty (N = 44)	p-value
Cervical (n = 17)	Axial pain			
	Preop	4.50 ± 2.55	3.50 ± 2.81	0.471
	1M	3.00 ± 2.54	2.50 ± 0.58	0.827
	6M	2.86 ± 3.29	2.75 ± 0.50	0.276
	1Y	2.83 ± 3.60	2.75 ± 0.50	0.181
	Delta(1M-1Y)	1.50 ± 2.07	−0.25 ± 0.50	0.104
	Radiating pain			
	Preop	3.40 ± 2.46	4.33 ± 3.83	0.546
	1M	2.78 ± 2.44	2.00 ± 1.41	0.749
	6M	1.86 ± 2.34	1.50 ± 0.58	0.602
	1Y	2.00 ± 2.53	1.50 ± 0.58	0.729
	Delta(1M-1Y)	2.00 ± 1.79	0.50 ± 1.73	0.377
Thoracic (n = 51)	Axial pain			
	Preop	3.71 ± 2.35	3.68 ± 2.52	0.741
	1M	2.00 ± 2.04	2.30 ± 1.18	0.340
	6M	1.15 ± 1.46	2.90 ± 1.89	0.002∗
	1Y	1.83 ± 1.75	2.90 ± 1.73	0.148
	Delta(1M-1Y)	0.75 ± 1.86	−0.62 ± 1.66	0.028∗
	Radiating pain			
	Preop	3.81 ± 2.36	4.84 ± 2.17	0.146
	1M	2.04 ± 2.36	2.26 ± 1.74	0.368
	6M	1.35 ± 1.93	1.24 ± 1.22	0.661
	1Y	1.83 ± 2.33	1.05 ± 1.40	0.363
	Delta(1M-1Y)	0.92 ± 1.00	1.29 ± 1.90	0.564
Lumbar (n = 31)	Axial pain			
	Preop	5.11 ± 3.23	4.50 ± 2.32	0.588
	1M	1.78 ± 1.40	2.92 ± 1.83	0.126
	6M	1.43 ± 1.28	1.91 ± 1.81	0.532
	1Y	1.89 ± 0.93	2.00 ± 1.89	0.800
	Delta(1M-1Y)	0.78 ± 1.56	0.80 ± 1.62	1.000
	Radiating pain			
	Preop	4.78 ± 2.94	4.70 ± 2.21	0.961
	1M	1.56 ± 1.46	2.42 ± 1.38	0.155
	6M	1.21 ± 1.31	1.18 ± 1.17	0.909
	1Y	1.67 ± 1.12	0.90 ± 1.20	0.107
	Delta(1M-1Y)	1.00 ± 1.73	1.60 ± 1.26	0.376

Values are presented as Mean ± standard deviation (SD). NRS, Numeric Rating Scale; Preop, preoperative; 1-month, 6-months, 1-year postoperatively (1M/6M/1Y).Delta 1M–1Y represents the change in NRS scores from 1 month to 1 year postoperatively.∗Statistically significant difference between groups (p < 0.05).

A recent systematic review and meta-analysis comparing hemilaminectomy and laminectomy for spinal tumors highlighted that most of the existing literature predominantly focuses on several parameters, such as total resection rates, neurological deterioration, postoperative complications, length of hospital stay, operative time, and estimated blood loss (Piñeiro et al., 2025). However, previous studies that attempted to evaluate long-term postoperative chronic pain have shown several critical limitations (Liao et al., 2023; Turel et al., 2015; Sun et al., 2011; Džurlić et al., 2026). Zong et al. demonstrated that heterogeneous follow-up durations across studies precluded objective comparisons (Zong et al., 2013). Furthermore, Dobran et al. measured pain scores over a relatively short follow-up period of only one month, which makes it difficult to adequately assess persistent postoperative axial pain (Dobran et al., 2021). In contrast, our current study introduces novelty by utilizing consistent follow-up intervals over a relatively prolonged evaluation period of one year, while specifically analyzing the differences in clinical outcomes based on the spinal level of the lesion.

The unilateral approach of hemilaminectomy minimizes trauma by restricting muscle dissection to one side and preserving midline structures like the spinous process and interspinous ligaments. In contrast, laminoplasty requires bilateral subperiosteal stripping and muscle detachment (Sun et al., 2011; He et al., 2022; Chen et al., 2024). Despite anatomical reconstruction with plates, the extensive muscular trauma and subsequent atrophy in the laminoplasty group likely contribute to the higher and more persistent axial pain observed (Wang et al., 2011; Hosono et al., 1996). Therefore, preserving unilateral musculoskeletal integrity is the decisive factor in enhancing long-term clinical recovery (Kameyama et al., 2023). A key finding of this study is that the clinical superiority of hemilaminectomy was most pronounced in the thoracic subgroup, while results in the cervical and lumbar regions were comparable between the two approaches. Our radiological analysis provides a plausible explanation for this result. As shown in Table 2, the thoracic spine exhibited significantly lower facet degeneration (0.59 ± 0.80) compared to the lumbar spine (1.09 ± 1.03; p = 0.018).

We hypothesize a “confounding influence” in the more mobile cervical and lumbar regions. Biomechanical and imaging studies consistently show a rostro-caudal gradient of degenerative change along the spinal column, with the lumbar region bearing the greatest load and demonstrating the highest prevalence and progression of disc and facet degeneration (Bernatz et al., 2024; Gellhorn et al., 2013). Imaging-based assessments further indicate relatively lower rates of facet osteoarthritis and paraspinal muscle fatty infiltration in thoracic segments compared with lumbar levels (Bernatz et al., 2024; Sollmann et al., 2020; Wang et al., 2015). Because these segments often harbor pre-existing degenerative changes, the baseline pain associated with such degeneration may overshadow the additional pain caused by surgical trauma. However, in the relatively degeneration-free thoracic segments with minimal pre-existing degeneration, the impact of the surgical technique becomes the dominant factor influencing postoperative pain. In these healthy segments, the structural preservation afforded by hemilaminectomy translates directly into superior clinical outcomes, whereas the bilateral trauma of laminoplasty results in a more noticeable increase in axial morbidity (He et al., 2020).

Concerns are often raised regarding whether limited access in hemilaminectomy compromises the extent of tumor resection. Our data showed that GTR achieved 100% of the hemilaminectomy group, comparable to the laminoplasty group. The only cases where GTR was not achieved were three instances of en plaque or broad-based meningiomas in the laminoplasty group. These cases likely represented more complex pathologies with significant dural adhesion, for which a wider bilateral corridor was intentionally selected. Our findings suggest that for the majority of standard IDEM tumors, the focused corridor of a hemilaminectomy is sufficient for safe and complete excision without compromising oncological outcomes.

Based on our findings, we consider unilateral hemilaminectomy to be a viable surgical option for thoracic IDEM tumors to minimize surgery-induced axial pain. By avoiding bilateral muscle trauma in segments with low baseline degeneration, surgeons can potentially enhance the quality of postoperative recovery.

This study has several limitations. First, its retrospective nature and the relatively small sample size in the cervical and lumbar subgroups may have limited the statistical power to detect differences in those regions. Second, the follow-up period of one year, while sufficient for observing recovery trends, may not capture very long-term structural changes. Third, our clinical assessment relied solely on the NRS for pain. Future research incorporating more comprehensive and multi-dimensional clinical outcome measures—such as the Oswestry Disability Index, Neck Disability Index, or EQ-5D—is necessary for a more diverse analysis of patient prognosis and functional recovery. Furthermore, although no definitive difference in tumor extent was observed between the two surgical groups, the operating surgeon's preference served as the determining factor for the surgical approach; thus, the possibility of selection bias cannot be completely excluded. In addition, different surgical and non-surgical protocols may affect pain progress outcome. Specifically, as a surgical aspect, the laminoplasty technique utilized in this study involves rigid reconstruction with a titanium mini-plate system, a method primarily employed in tumor resection to restore the spinal canal (Chen et al., 2024). In contrast, standard laminectomy for degenerative spinal disease often does not involve such reconstruction (Piñeiro et al., 2025). This difference in surgical protocol may limit the direct comparability of our results with broader degenerative surgical outcomes. Our result indicates a slight exacerbation of axial pain was observed in the laminoplasty group between 1 month and 1 year postoperatively. As a non-surgical aspect, we speculate that this finding may be primarily attributed to our institutional protocol, which involves the routine discontinuation of postoperative bracing and pain medications at one month.

Finally, while hemilaminectomy is a muscle-preserving approach, future studies comparing this technique with endoscopic surgery and the application of robotic-assisted systems, such as the Da Vinci Robot—which further minimizes paraspinal muscle disruption—could help identify even more optimized surgical strategies for IDEM tumors (Choucha et al., 2025). Although its current application remains selective, as endoscopic techniques evolve and a robust evidence base for pain reduction is established, they could potentially offer a definitive pathway to further mitigating chronic surgery-induced axial pain by achieving the ultimate goal of minimal tissue trauma (Dhandapani and Gendle, 2024; Zhang et al., 2023). Prospective studies with larger cohorts across all spinal regions are warranted to further validate the relationship between baseline degeneration and surgical outcomes.

## 5 Conclusion

Unilateral hemilaminectomy is an effective and muscle-preserving technique for the resection of IDEM tumors. It provides superior long-term axial pain relief compared to laminoplasty, particularly in the thoracic spine. This level-specific advantage is likely due to the lower baseline degeneration in thoracic segments, which makes the clinical benefits of structural preservation more apparent.

## Declaration of competing interest

The authors declare the following financial interests/personal relationships which may be considered as potential competing interests: Hyun-Jun Jang reports was provided by Department of Neurosurgery, Spine and Spinal Cord Institute, Gangnam Severance Hospital, Yonsei University College of Medicine, Seoul, Korea. Hyun-Jun Jang reports a relationship with Department of Neurosurgery, Spine and Spinal Cord Institute, Gangnam Severance Hospital, Yonsei University College of Medicine, Seoul, Korea that includes:. If there are other authors, they declare that they have no known competing financial interests or personal relationships that could have appeared to influence the work reported in this paper.
